# EMG changes during continuous intraoperative neuromonitoring with sustained recurrent laryngeal nerve traction in a porcine model

**DOI:** 10.1007/s00423-016-1419-y

**Published:** 2016-04-16

**Authors:** Katrin Brauckhoff, Turid Aas, Martin Biermann, Paul Husby

**Affiliations:** 1 Department of Endocrine Surgery, Haukeland University Hospital, Jonas Lies vei 65, N-5021 Bergen, Norway; 2 Department of Clinical Medicine, University of Bergen, Bergen, Norway; 3 Department of Anesthesia and Intensive Care, Haukeland University Hospital, Bergen, Norway

**Keywords:** Thyroid surgery, Continuous neuromonitoring, Recurrent laryngeal nerve, Vocal cord palsy, Experiment

## Abstract

**Purpose:**

Traction is the most common cause of injury to the recurrent laryngeal nerve (RLN) in endocrine neck surgery. The purpose of this study was to evaluate specific alterations to the electromyogram (EMG) and verify safe alarm limits in a porcine model of sustained traction of the RLN using continuous intraoperative neuromonitoring (C-IONM).

**Methods:**

Sixteen Norwegian Landrace pigs were anesthetized and intubated with a tracheal tube with a stick-on laryngeal electrode. EMG was recorded at baseline (BL) and during sustained traction applied to each RLN until 70 % amplitude decrease from BL, and during 30-min recovery.

**Results:**

In 29 nerves at risk (NAR), BL amplitude and latency values were 1098 ± 418 (586–2255) μV (mean ± SD (range)) (right vagus) and 845 ± 289 (522–1634) μV (left vagus), and 4.7 ± 0.5 (4.1–5.9) ms and 7.9 ± 0.8 (6.7–9.6) ms, respectively. At 50 % amplitude decrease, latency increased by 14.0 ± 5.7 % (right side) and 14.5 ± 9.1 % (left side) compared with BL. Corresponding values for 70 % amplitude depression were 17.9 ± 6.1 % and 17.3 ± 12.8 %. Traction time to 50 and 70 % amplitude decrease ranged from 3 to 133 min and 3.9–141 min, respectively. In 16 NAR (55 %), time from 50 to 70 % reduction in amplitude was ≤5 min, but in six NAR (21 %) ≤1 min. In only 11 (38 %) of 29 nerves, the amplitude recovered to more than 50 % of BL.

**Conclusions:**

Latency increase may be the first warning of RLN stretch injury. Given the short interval between 50 and 70 % amplitude reduction of the EMG, amplitude reduction by 50 % can be taken as an appropriate alert limit.

## Introduction

Injury to the recurrent laryngeal nerve (RLN) resulting in postoperative vocal fold palsy is the most common cause for impaired quality of life after endocrine neck surgery [1–3]. The incidence ranges from 2.6 to 26 %, depending on the diagnosis, surgical procedure, and the surgeon’s skills [1, 4]. Routine visual identification of the RLN has been associated with decreased incidence of postoperative vocal fold palsies [5]. Bilateral RLN injury, the most dreaded complication, can be avoided by intraoperative neuromonitoring. Neuromonitoring predicts postoperative vocal fold function and allows changing operative strategy to a two-stage thyroidectomy if necessary [6, 7]. Unilateral postoperative vocal fold palsy rates have, however, not been reduced by intermittent intraoperative neuromonitoring (I-IONM) [1, 4, 8].

The most common cause of intraoperative RLN injury is traction to the nerve [9, 10]. Traction can cause acute and localized damage to the nerve leading to loss of signal (LOS) or evolve over time with gradual impairment of nerve function. Most often, I-IONM detects a nerve lesion only after the injury is manifest as LOS [11]. In contrast, continuous intraoperative neuromonitoring (C-IONM) controls the integrity of the nerve nearly in real time. Warning to the surgeon can be given timely enough to cease a harmful maneuver and thereby avoid complete nerve lesion [12, 13].

C-IONM may potentially reduce the risk for intraoperative nerve injury caused by harmful stress over time such as sustained or repetitive traction. This requires that imminent damage to the nerve can be reliably identified by specific changes to the electromyogram (EMG) which are easy to distinguish from artifacts caused by electrode dislocation. A recent retrospective clinical study has shown that combined EMG events defined as concurrent amplitude reduction >50 % and latency increase by >10 % predict LOS if the harmful stress is not eliminated by the surgeon [13].

Aiming for a more thorough validation of the currently accepted alert limits during C-IONM using a commercial C-IONM system, we studied EMG changes in a porcine model of sustained traction injury to the RLN.

## Materials and methods

### Animals, animal handling, and anesthesia

Sixteen immature domestic pigs (Norwegian Landrace, Norhybrid; age 3 months) of either sex were acclimatized for at least 1 week in our laboratory housing area. Anesthetic and experimental protocols were approved by the local animal veterinarian (Vivarium, University of Bergen, Norway) under surveillance of the Norwegian Animal Research Authority, Oslo, Norway and in accordance with current regulations [14]. Food was withdrawn 12 h prior to the experiments, whereas water was available at all times.

Thirty minutes after intramuscular administration of preanesthetic medication (atropine 1 mg, diazepam 10 mg, ketamine 500 mg), general anesthesia was induced via a face mask with isoflurane in oxygen. An intravenous (i.v.) catheter was placed in an ear vein, and anesthesia supplemented with thiopentone (5 mg/kg body weight) i.v. After 2 min, tracheal intubation was then performed using a Lo-Contour oral/nasal tracheal tube (internal diameter 6.5 or 7.0 mm; Mallinckrodt™, Covidien, Mansfield, MA), supplemented with a Dragonfly “wrap-around” single-channel laryngeal electrode for C-IONM (Spes Medica S.r.l., Genova, Italy). General anesthesia was maintained by volume-controlled ventilation (Dräger anesthesia workstation, Dräger, Lübeck, Germany) with isoflurane delivered in 50 % oxygen in air and a continuous i.v. infusion of fentanyl (7.5 μg/kg/h) and midazolam (0.5 mg/kg/h) [15]. Adjustment of the inspired isoflurane concentration was allowed in the range of 0.5–2.0 vol.% according to reactions to standardized noxious stimuli [16]. No neuromuscular blocking agents were allowed. End-tidal carbon dioxide level was maintained about 5 kPa. Body core temperature was stabilized by a heating mattress and covering blankets.

At the end of each experiment, the pigs were sacrificed by an i.v. injection of 20 ml of saturated solution of potassium chloride.

### Surgical preparation

**Fig. 1:**
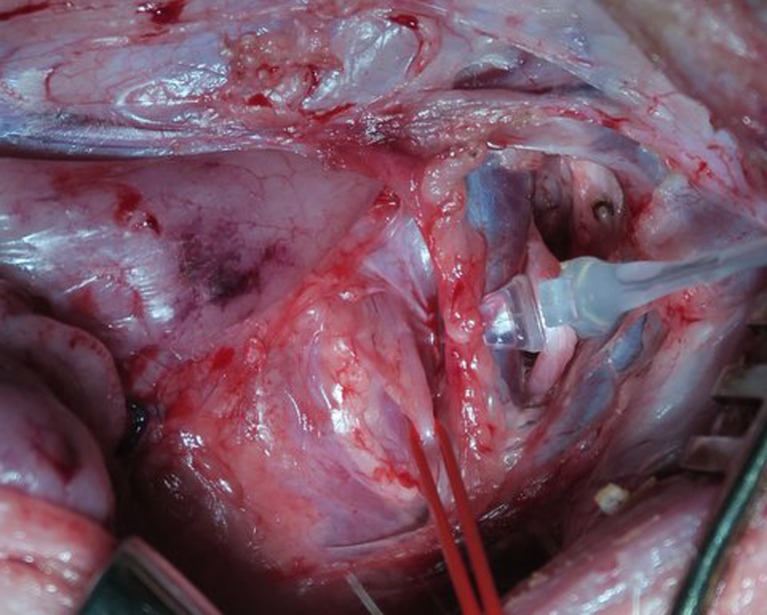
Surgical site in an experimental animal. The left vagal nerve is being stimulated with a saxophone electrode (*gray*). Traction is being applied to the left recurrent laryngeal nerve by means of a vessel loop (*red*)

Neck and larynx were exposed by a vertical collar incision 2 cm above the sternum. The vagus nerve was identified visually and with the use of a bipolar handheld stimulation probe (4 Hz, 200 μs, 1 mA; Dr. Langer; Dr. Langer Medical GmbH, Waldkirch, Germany). Thereafter, a Dr. Langer Saxophone electrode (3 Hz, 200 μs, 1 mA) was carefully placed on the vagal nerve. The ipsilateral RLN was identified by means of magnifying glasses and a handheld probe, and a vessel loop gently wrapped around the nerve at the level of the fourth tracheal ring (Fig. 1). Preparation was kept to a minimum in order to preserve all connective tissue around the nerve. Before each registration, the position of the tracheal tube was readjusted to obtain the optimal amplitude at BL. After BL EMG recording, traction was initiated by attaching a plumb system weighting 108 g in total to the vessel loop via a pulley, yielding a constant force of 1.06 N as used in the experiments by Lee to avoid acute damage of the nerve.[17] Traction was discontinued when the EMG amplitude decreased 70 % from baseline. After traction was terminated, RLN mapping was performed with the handheld probe (I-IONM) and the point of injury localized. The nerve was allowed to recover for 30 min under C-IONM registration. The right and left sides were studied in reverse order in every second animal. The contralateral side was exposed after completion of all measurements on the first side. During traction, no further manipulation was undertaken. The EMG was recorded continuously from BL until the end of recovery using the Dr. Langer AVALANCHE™ system. Loss of signal (LOS) was defined as an amplitude below 100 μV. In the 16 animals with 32 nerves at risk (NAR), three NAR showed LOS before start of traction due to acute irreversible injury during preparation and were excluded from analysis.

### Hemodynamic monitoring

Heart rate (HR) was monitored using surface ECG electrodes. Systemic mean arterial pressure (MAP) and central venous pressure (CVP) were followed by fluid-filled catheters introduced into the right femoral artery and vein, connected to pressure transducers (Transpac™, ICU Medical, San Clemente, CA) connected to an IntelliVue monitor (Philips, Böblingen, Germany).

### Statistics

Data were maintained in an MDCake client–server database and analyzed using the statistics program “R” version 3.2.1. [18, 19]. Analysis of Variance (ANOVA) was used to test for differences in physiological parameters. Time differences to 50 and 70 % amplitude reduction were evaluated by unpaired *t* test. Significance level was set as *p* < 0.05 (two-sided).

## Results

Sixteen animals aged 93 ± 10 days (mean ± SD), 50 % female and weighing 40 ± 4 kg were studied. All animals remained stable with respect to HR, MAP, CVP, and other physiological parameters. Body core temperature was 38.0 ± 0.6 ^○^C at the start and 38.5 ± 0.7 ^○^C at the end of the experiments (n.s.).

### EMG parameters

**Fig. 2:**
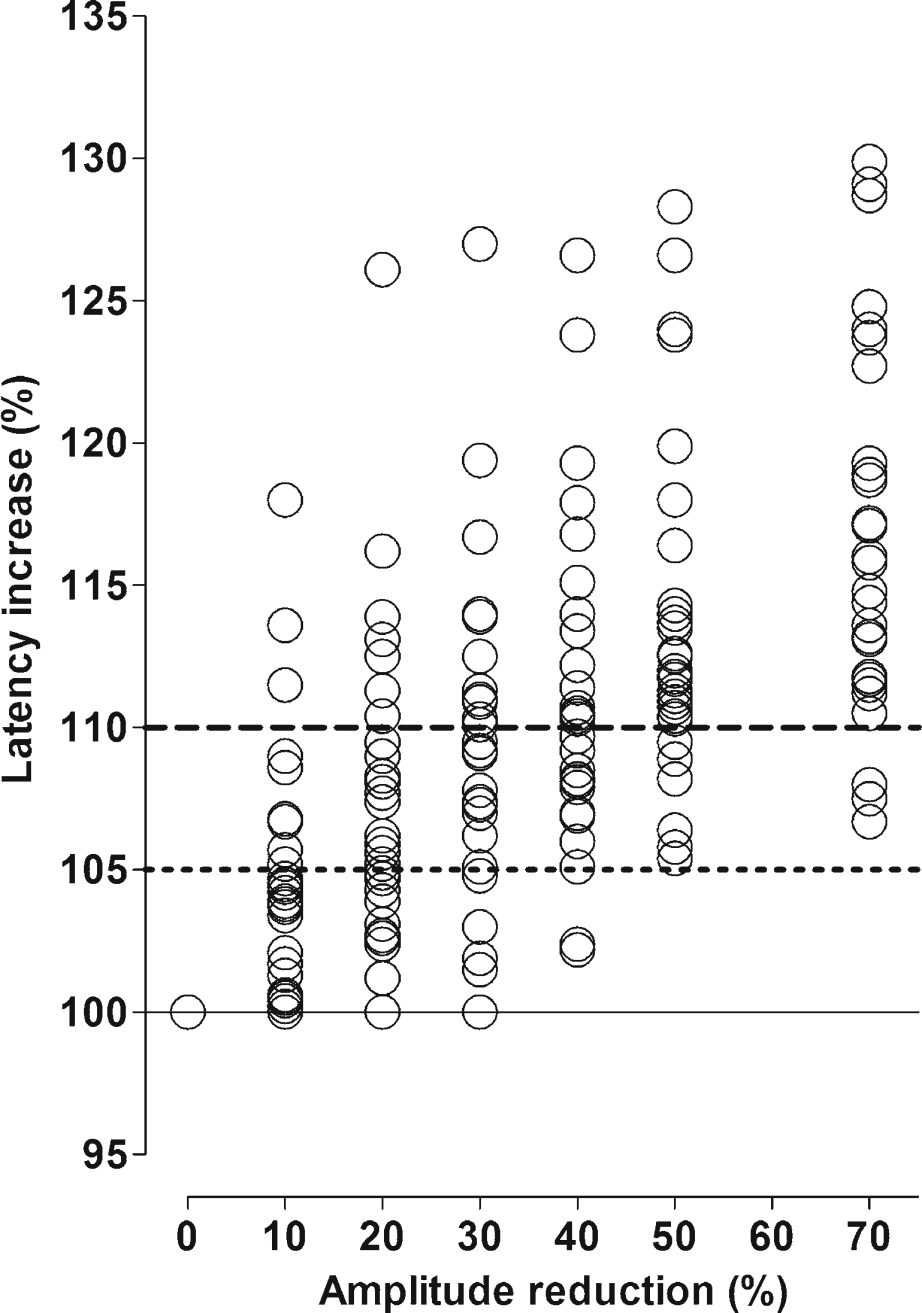
Scatterplot of amplitude and latency changes following sustained traction applied to the recurrent laryngeal nerve in all 29 nerves. Amplitude changes are depicted at 10, 20, 30, 40, 50 and 70 % amplitude reduction below baseline with corresponding latency values

BL amplitudes following stimulation of the right and left vagal nerve were 1098 ± 418 (586–2255) μV (mean ± SD (range)) and 845 ± 289 (522–1634) μV, respectively. Amplitudes tended to be higher on the right than left side (*p* = 0.07). BL latency was 4.7 ± 0.5 (4.1–5.9) ms on the right side and 7.9 ± 0.8 (6.7–9.6) ms on the left side (*p* < 0.001). During sustained traction applied to the RLN, latency increased to ≥110 % in 22 (76 %) of 29 nerves before amplitude decreased to 50 % of BL as depicted in Fig. 2 and Table 1. As amplitude decreased to 30, 40, and, 50 % from BL, we observed latency increases to 109.4 ± 5.6 % (mean ± SD); 111.2 ± 5.7 % and 114.2 ± 7.3 %, respectively. The injury point was always found at the area where traction was applied to the nerve (Type 1- injury).

**Table 1:** Time course of electromyographic signal changes during and 30 min after sustained traction of the recurrent laryngeal nerve

Animal/ Side	Baseline EMG		Time to L110% (min)	Time to A50% (min)	Time to A70% (min)	Lowest A after TR		30 min after TR	
	A (μV)	L (ms)				A_min_ (μV)	L (ms)	A (μV)	L (ms)
1/R	1169	4.58	11	12	13	312	5.18	412	4.98
2/L	522	6.76	5	6	7	99	7.56	123	7.16
2/R	908	4.24	27	23	37	272	4.74	468	4.42
3/L	741	8.28	5	23	25	0		324	9.44
3/R	695	4.12	39	48	53	233	5.32	276	4.56
4/L	5224	6.66	40	65	76	123	7.18	150	8.884
4/R	2255	4.28	24	101	131	300	4.76	781	4.60
5/L	1634	8.20	32	29	32	448	9.06	473	8.68
5/R	1052	4.86	20	19	24	281	5.44	957	5.20
6/L	784	7.822	29	34	46	165	8.74	287	8.50
6/R	1873	4.50	2	2	2	358	4.70	573	4.78
7/L	838	8.42	64	38	64	223	9.04	300	8.58
7/R	1016	4.58	29	37	47	208	5.10	708	4.88
8/L	1175	8.36	124	130	135	96	10.60	396	8.64
8/R	1056	4.60	139	133	141	309	5.46	724	4.66
9/R	586	5.92	12	14	16	114	7.16	206	6.35
10/R	949	5.16	19	30	38	196	6.24	459	5.54
11/L	593	9.64	X	39	63	169	10.40	265	10.34
11/R	966	5.30	6	8	8	284	6.30	627	5.54
12/L	1017	8.26	19	55	60	330	10.22	883	9.90
12/R	1229	4.76	5	43	51	343	6.00	911	5.30
13/L	822	8.08	4	5	6	169	8.82	360	8.46
13/R	1124	4.88	10	50	53	244	5.78	577	5.25
14/L	808	7.76	12	13	17	233	8.72	484	8.08
14/R	1023	5.12	9	14	16	210	5.48	512	5.40
15/L	902	7.68	47	42	74	271	8.58	295	8.02
15/R	842	4.42	24	38	49	230	5.13	341	4.60
16/L	692	7.36	42	75	88	194	8.52	274	7.56
16/R	825	4.44	3	4	4	259	5.20	521	4.72

*A* amplitude, *L* latency, *TR* traction, *L110%* latency increase to 110 % of baseline, *A50% and A70%* amplitude to decrease 50 and 70 % of baseline, *X* L110% not reached

**Fig. 3:**
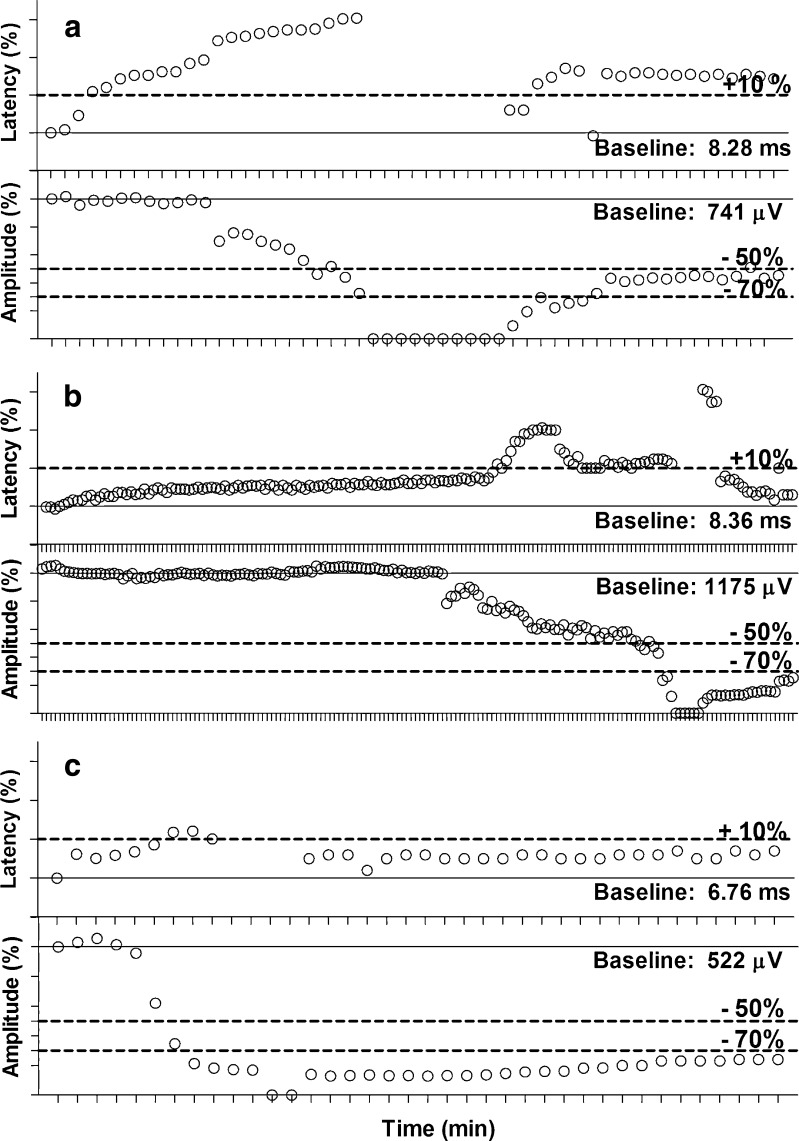
EMG changes as function of time during sustained traction applied to the recurrent laryngeal nerve in three nerves (**a**, **b**, **c**), that progressed to LOS after traction release at an amplitude reduction of 70 % from baseline. Values are given for every 60 s during traction and 30 min recovery

Immediately after release of traction, amplitude was 230 ± 93 (0–448) μV. After a 30-min recovery, it increased to 472 ± 468 (123–957) μV, remaining 52 ± 18 (9–76) % below BL. The amplitude recovered to more than 50 % of BL only in 11 (38 %) of 29 nerves. In three NAR (10.3 %), the EMG changes progressed to LOS following release of traction. The time-related and detailed EMG changes in these nerves are presented in Fig. 3.

Traction times until 50 and 70 % amplitude reduction were highly variable (Table 1, Fig. 3). Of note, the time interval from 50 to 70 % amplitude reduction was 5 min or less in 16 NAR (55 %) and 1 min or less in six NAR (21 %).

All the registered waveforms were biphasic, and artificial changes in waveform were not observed.

## Discussion

In contrast to I-IONM, C-IONM has the potential to recognize threatening nerve lesions by monitoring nerve function nearly in real time. This potential is limited by the time span in which the nerve injury appears. When injuries develop over time—such as traction injury or compression of the nerve—the surgeon is given the chance to release the stressing maneuver and thus avoid permanent damage to the nerve.

Traction is the most common cause of RLN injury in about 80 % [9]. Sustained or repeated traction to the RLN can result in nerve injury [20]. Studies in humans have shown that impending nerve injury can be recognized by EMG changes as combined events affecting amplitude and latency [12, 13]. Schneider et al. defined mild combined events as 50–70 % decrease in amplitude with a 5–10 % increase in latency, intermediate combined events with amplitude >50 % decrease and latency >10 % increase, and severe combined events with >70 % amplitude decrease and latency >10 % increase.

To study EMG changes under sustained traction on the RLN, we chose a porcine model. Several studies has shown that the porcine anatomy and electrophysiology of the RLN and vagus nerve are comparable with humans [20, 21]. EMG changes due to artifacts by dislocation of the EMG-tube or poor contact with the tube electrode have been described as amplitude changes without any latency alteration [22]. In our study, we eliminated artifacts by avoiding manipulation of the surgical field after initial preparation.

Harm to the RLN resulting in neuropraxia or axonotmesis depends on a combination of harm power and harm duration. In this study, traction force was kept constant. Alterations in amplitude and latency developed gradually over time. Traction times until 70 % amplitude reduction varied considerably between animals, but also between the two nerves in the same animal. We were not able to identify a direct cause to this variation. Similar intra- and interindividual variation was recently reported in a comparable porcine model of sustained traction injury to the RLN (traction force 1.2 N) [23].

In our experiments, latency increases to 105 and 110 % preceded amplitude decrease to 50 %. In an experimental study by Lee et al., four nerves exposed to traction injury showed an increase in EMG latency, but no amplitude reduction [17]. Latency is defined as the time from the electric impulse given to the vagus nerve to the start of the muscle contraction in the vocal cord. It is a function of nerve conduction velocity, distance along the nerve and the conduction delay across the neuromuscular junction. In our experimental model, the RLN was elongated by 3–5 mm under traction. Assuming a constant nerve conduction velocity between 50 and 70 m/s, a nerve elongation of 3–5 mm will increase latency by no more than 0.04–0.1 ms. This implies that the alteration of nerve conduction is rather the effect of structural or functional changes in the myelin sheath or by the surrounding epi- and perineurium. Wu et al. found distortion of the structure in the perineurium and epineurium in traction-injured nerve fibers, whereas Lee et al. could not find any visual signs of injury to the nerve [17, 20]. We suggest that early latency increase in sustained traction injuries may be a consequence of altered epineurium and perineurium that affect the isolation of the nerve and reduce nerve conduction before the myelin sheath or axon are affected.

We found that in 55 % (16) NAR the time interval between 50 and 70 % amplitude reduction was only 5 min or less and in 21 % (6) 1 min or less. Furthermore, amplitude continued to decrease even after the release of traction and proceeded to LOS occurring in three nerves. These data imply that the 50 % amplitude reduction, but not 70 %, is a safe limit to prevent LOS. Schneider et al. found recovery to 50 % of the BL amplitude was always accompanied with normal postoperative vocal cord function [10]. However, when the amplitude recovered to less than 50 % of BL, postoperative vocal cord function was impaired in 100 % of patients with type 1 LOS (localized injury point in the extralaryngeal course of the RLN) and in 67 % of the patients with type 2 LOS (global). In our study, all nerves showed signs of recovery, but in 62 % (18) NAR, 50 % of initial BL amplitude were not reached within 30 min of recovery. None of the nerves with LOS recovered over the 50 % amplitude level.

Our animal study has several limitations. First, an animal model may not extend to human anatomy and pathophysiology. Second, the injury mechanism is simplified compared to conditions during thyroid surgery in humans where traction can occur with different forces and both acutely and repetitively. Third, the EMG changes were not correlated with postoperative functional examination of the vocal cord. Fourth, we investigated only type 1 injury. It remains unclear if our data can provide valid insights into global type 2 injury.

## Conclusion

In an experimental porcine model of traction injury to the RLN by means of a constant force, increase in EMG latency to 110 % of baseline preceded 50 % amplitude reduction and may, thus, be a first warning sign of impending nerve lesion. The 50 % amplitude limit appears to be an appropriate criterion to avoiding nerve damage due to traction in endocrine neck surgery.

## References

[1] Dralle H, Sekulla C, Lorenz K, Brauckhoff M, Machens A, German IONM Study Group (2008). Intraoperative monitoring of the recurrent laryngeal nerve in thyroid surgery. World J Surg.

[2] Dralle H, Lorenz K, Machens A (2012). Verdicts on malpractice claims after thyroid surgery: emerging trends and future directions. Head Neck.

[3] Lydiatt DD (2003). Medical malpractice and the thyroid gland. Head Neck.

[4] Randolph GW, Dralle H, Abdullah H, Barczynski M, Bellantone R, International Intraoperative Monitoring Study Group (2011). Electrophysiologic recurrent laryngeal nerve monitoring during thyroid and parathyroid surgery: international standards guideline statement. Laryngoscope.

[5] Dralle H, Sekulla C, Haerting J, Timmermann W, Neumann HJ, Kruse E (2004). Risk factors of paralysis and functional outcome after recurrent laryngeal nerve monitoring in thyroid surgery. Surgery.

[6] Dralle H, Sekulla C, Lorenz K, Nguyen Thanh P, Schneider R, Machens A (2012). Loss of the nerve monitoring signal during bilateral thyroid surgery. Br J Surg.

[7] Melin M, Schwarz K, Lammers BJ, Goretzki PE (2013). IONM-guided goiter surgery leading to two-stage thyroidectomy—indication and results. Langenbecks Arch Surg.

[8] Barczyński M, Konturek A, Cichoń S (2009). Randomized clinical trial of visualization versus neuromonitoring of recurrent laryngeal nerves during thyroidectomy. British J Surg.

[9] Chiang F-Y, Lu I-C, Kuo W-R, Lee K-W, Chang N-C, Wu C-W (2008). The mechanism of recurrent laryngeal nerve injury during thyroid surgery—the application of intraoperative neuromonitoring. Surgery.

[10] Schneider R, Sekulla C, Machens A, Lorenz K, Thanh PN, Dralle H (2015) Dynamics of loss and recovery of the nerve monitoring signal during thyroidectomy predict early postoperative vocal fold function. Head & Neck. doi:10.1002/hed.24175 10.1002/hed.24175 26331940

[11] Dralle H, Lorenz K, Schabram P, Musholt TJ, Dotzenrath C, Goretzki PE, Kuβmann J, Niederle B, Nies C, Schabram J, Scheuba C, Simon D, Steinmüller T, Trupka A, et al (2013) Intraoperative neuromonitoring in thyroid surgery. Recommendations of the Surgical Working Group for Endocrinology. Chirurg 84:1049–1056. doi:10.1007/s00104-013-2656-z 10.1007/s00104-013-2656-z 24337220

[12] Phelan E, Schneider R, Lorenz K, Dralle H, Kamani D, Potenza A, Sritharan N, Shin JW, Randolph G (2014). Continuous vagal IONM prevents recurrent laryngeal nerve paralysis by revealing initial EMG changes of impending neuropraxic injury: a prospective, multicenter study. Laryngoscope.

[13] Schneider R, Randolph GW, Sekulla C, Phelan E, Thanh PN, Bucher M, Machens A, Dralle H, Lorenz K (2013). Continuous intraoperative vagus nerve stimulation for identification of imminent recurrent laryngeal nerve injury. Head Neck.

[14] Institute of Laboratory Animal Resources, National Research Counci (2011). Guide for the care and use of laboratory animals. 8. edn 2. printing.

[15] Husby P, Heltne JK, Koller ME, Birkeland S, Westby J, Fosse R, Lund T (1998). Midazolam-fentanyl-isoflurane anaesthesia is suitable for haemodynamic and fluid balance studies in pigs. Lab Anim.

[16] Boschert K, Flecknell PA, Fosse RT, Framstad T, Ganter M, Sjøstrand U, Stevens J, Thurman J (1996). Ketamine and its use in the pig. Recommendations of the Consensus meeting on Ketamine Anaesthesia in Pigs, Bergen 1994. Ketamine Consensus Working Group. Lab Anim.

[17] Lee HY, Cho YG, You JY, Choi BH, Kim JY, Wu C-W, Chiang F-Y, Kim HY (2014). Traction injury of the recurrent laryngeal nerve: Results of continuous intraoperative neuromonitoring in a swine model. Head Neck.

[18] Biermann M, Kråkenes J, Brauckhoff K, Haugland HK, Heinecke A, Akslen LA, Varhaug JE, Brauckhoff M (2015). Post-PET ultrasound improves specificity of 18F-FDG-PET for recurrent differentiated thyroid cancer while maintaining sensitivity. Acta Radiol.

[19] Biermann M (2014). A simple versatile solution for collecting multidimensional clinical data based on the CakePHP web application framework. Comput Methods Programs Biomed.

[20] Wu C-W, Dionigi G, Sun H, Liu X, Kim HY, Hsiao P-J (2014). Intraoperative neuromonitoring for the early detection and prevention of RLN traction injury in thyroid surgery: a porcine model. Surgery.

[21] Lorenz K, Sekulla C, Schelle J, Schmeiss B, Brauckhoff M, Dralle H, German Neuromonitoring Study Group (2010). What are normal quantitative parameters of intraoperative neuromonitoring (IONM) in thyroid surgery?. Langenbecks Arch Surg.

[22] Kim HY, Tufano RP, Randolph G, Barczyński M, Wu C-W, Chiang F-Y (2015). Impact of positional changes in neural monitoring endotracheal tube on amplitude and latency of electromyographic response in monitored thyroid surgery: results from the porcine experiment. Head Neck.

[23] Béchu M, Lauzana E, Köhler P, Klein S, Rashid N, Kahle E, Meyding-Lamadé U, Lamadé W (2015). Inter- and intraindividual differences of vulnarability of recurrent laryngeal nerves under tensile stress in a porcine model. J Neurol Sci.

